# Guidelines for Robotic Flexible Endoscopy at the Time of COVID-19

**DOI:** 10.3389/frobt.2021.612852

**Published:** 2021-02-25

**Authors:** Onaizah Onaizah, Zaneta Koszowska, Conchubhair Winters, Venkatamaran Subramanian, David Jayne, Alberto Arezzo, Keith L. Obstein, Pietro Valdastri

**Affiliations:** ^1^ School of Electronic and Electrical Engineering, University of Leeds, Leeds, United Kingdom; ^2^ Leeds Institute of Medical Research, University of Leeds, Leeds, United Kingdom; ^3^ Department of Surgical Sciences, University of Torino, Torino, Italy; ^4^ Department of Gastroenterology, Hepatology, Nutrition, Vanderbilt University Medical Center, Nashville, TN, United States; ^5^ Department of Mechanical Engineering, Vanderbilt University, Nashville, TN, United States

**Keywords:** robotic flexible endoscopy, endoscopes, gastrointestinal, infection control, aerosol generating procedure, COVID-19

## Abstract

Flexible endoscopy involves the insertion of a long narrow flexible tube into the body for diagnostic and therapeutic procedures. In the gastrointestinal (GI) tract, flexible endoscopy plays a major role in cancer screening, surveillance, and treatment programs. As a result of gas insufflation during the procedure, both upper and lower GI endoscopy procedures have been classified as aerosol generating by the guidelines issued by the respective societies during the COVID-19 pandemic—although no quantifiable data on aerosol generation currently exists. Due to the risk of COVID-19 transmission to healthcare workers, most societies halted non-emergency and diagnostic procedures during the lockdown. The long-term implications of stoppage in cancer diagnoses and treatment is predicted to lead to a large increase in preventable deaths. Robotics may play a major role in this field by allowing healthcare operators to control the flexible endoscope from a safe distance and pave a path for protecting healthcare workers through minimizing the risk of virus transmission without reducing diagnostic and therapeutic capacities. This review focuses on the needs and challenges associated with the design of robotic flexible endoscopes for use during a pandemic. The authors propose that a few minor changes to existing platforms or considerations for platforms in development could lead to significant benefits for use during infection control scenarios.

## Endoscopy During COVID-19

### COVID-19

On March 11^th^, the WHO (World Health Organization) declared COVID-19 caused by the SARS-CoV-2 virus a pandemic. There have been over 94 million cases reported and over two million fatalities worldwide (Johns Hopkins University Coronavirus Center, 2020) as of January 2021. The main symptoms include fever, cough, change in smell or taste, breathlessness, and weakness with many people also reporting gastrointestinal symptoms such as abdominal pain, diarrhoea and vomiting. A small percentage develop acute respiratory distress syndrome (ARDS) which can be fatal. Human-to-human transmission primarily occurs through direct contact or droplets (Repici et al., 2020), with smaller droplets (often called aerosols) having the potential to remain airborne for an extended period of time and thus travel much larger distances. This means they cannot be tackled simply by physical distancing measures employed whereas larger particles are immediately pulled down due to gravity and risks can be mitigated with physical distancing measures.

Endoscopy is considered a high-risk procedure due to the proximity of health care workers (HCW) to patients and the potential for aerosol generation. Recent work has shown that endoscopists without proper face protection such as a face visor could be at an increased risk to bacterial pathogens (Johnston et al., 2019). Many studies have since shown that a face visor is also not adequate face protection from droplets (Akagi et al., 2020). Studies from the SARS-CoV-2 outbreak have shown that droplets could easily reach 6 ft (∼2 m) from an infected patient thereby putting HCW in endoscopy units at risk (Wong et al., 2004).

### Conventional Endoscopy

Endoscopy is a procedure where organs and tissues inside the body can be imaged and monitored using an endoscope. An endoscope is a thin tube with a light source and camera, often with additional tools, for example ultrasound or a working channel for introduction of biopsy forceps or therapeutic equipment. Endoscopy can be used for diagnostic (visualisation and sampling) and therapeutic purposes such as removing cancer tissue. An endoscope can be either rigid or flexible; with flexible endoscopes offering a multitude of advantages for navigation to target sites. Endoscopes can be inserted through natural orifices like the mouth, anus or urethra or via incisions made in the body. Flexible endoscopy is often used as a diagnostic tool for many types of cancer and diseases and thus plays a vital role in the management of multiple malignancies. There are many different types of endoscopy from bronchoscopy (monitoring the lungs) to hysteroscopy (monitoring the uterus) and cystoscopy (monitoring the bladder); however, for the purposes of this review we will limit our focus to flexible gastrointestinal (GI) endoscopy. There are a range of flexible GI endoscopies including esophagogastroduodenoscopy (EGD–for assessing esophagus, stomach, and duodenum), colonoscopy (for assessing the large bowel), sigmoidoscopy (for the rectum and sigmoid colon), endoscopic retrograde cholangiopancreatography (ERCP–for assessing the biliary tree and pancreatic ducts), and enteroscopy (for assessing the small intestine).

Colorectal cancer is the third most common cancer in the world in terms of mortality and fourth most common in terms of incidence reaching nearly two million cases and one million fatalities in 2018 according to some projections (Rawla et al., 2019). Colonoscopy can detect and remove pre-cancerous tissue in the colon, thus preventing the development of colorectal cancers. GI endoscopies are also the gold standard investigative method in the diagnosis and surveillance of a large variety of conditions such as celiac disease, and inflammatory bowel diseases. In 2014, it was projected that there will be over 75 million gastrointestinal endoscopic procedures performed by 2020 in Europe and the United States alone (Lau, 2014). There were more than two million total GI procedures in the United Kingdom in 2019 (Ravindran et al., 2020) and over 17 million total GI procedures in the United States in 2013 (Peery et al., 2019).

Conventional endoscopy uses a semi-rigid tube and manoeuvring the endoscope is performed manually by rotation of a set of wheels on the handle and by pushing, pulling, and torqueing the insertion tube of the endoscope. Many procedures are uncomfortable or painful, requiring analgesia and sedation. A typical GI endoscopy process requires multiple HCW inside the room–an endoscopist, an assistant/technician, and a nurse to monitor the patient. For general anaesthetic and fluoroscopic procedures, this could potentially include anaesthesiologists and radiographers. Typical pre-pandemic personal protective equipment (PPE) for these processes consisted of gloves, gown/apron, and eye protection.

### COVID-19 Related Risks During Flexible GI Endoscopy

Some endoscopic procedures are considered aerosol generating procedures (AGPs). Aerosols are small particles/droplets below 5 µm that can remain airborne for an extended period of time. One of the postulated sources of aerosol generation during endoscopy procedures is related to gas insufflation. Positive insufflation is used to visualize the lumen and create space to move the instrument forward. The potential generation of aerosols during endoscopy could pose a risk to HCW.

Evidence of aerosols generated during different endoscopic procedures varies and there is no homogeneity of evidence. Endoscopic procedures such as bronchoscopy have been shown to be aerosol generating along with several other patient care and operating room procedures (Mittal et al., 2020; Thamboo et al., 2020; Wahidi et al., 2020). However, for GI endoscopic procedures, no current evidence exists of aerosol generation and advice from respective societies around infection control (IC) is based on expert opinion (Chai et al., 2020; Mahadev et al., 2020; Repici et al., 2020; Tse et al., 2020). A well-designed study is needed to address this knowledge gap in the field that would allow tailored advice for specific endoscopic procedures.

Upper GI procedures are considered a greater risk during the current pandemic because the virus has been shown to be transmissible through airway secretions. The risk for lower GI procedures (e.g. colonoscopy and sigmoidoscopy) is less clear, although SARS studies have shown the presence of coronavirus in stool samples and in intestinal biopsy samples (Isakbaeva et al., 2004; Pan et al., 2020) and there is some data regarding the dispersion of microorganisms throughout an endoscopy suite during colonoscopy (Vavricka et al., 2010). There has also been some focus on identifying a COVID-19 outbreak through wastewater at several institutions in the United States, which would suggest either the presence of the virus or viral RNA in stool samples (Cahill and Morris, 2020). For now, most guidelines from gastroenterology societies have classified all GI endoscopic procedures as AGPs (Chai et al., 2020; Mahadev et al., 2020; Repici et al., 2020; Tse et al., 2020). Therefore, PPE has been enhanced during the COVID-19 pandemic to include a full sleeve gown, an FFP3/N95 mask, gloves, face visor or goggles, shoe covers, and a surgical hair cap.

### Potential for Innovation

Despite significant advances in the imaging capabilities of endoscopes, the controls remain largely unchanged in the last 60 years. The rear steering approach of conventional colonoscopy for example stretches the bowel and surround mesentery which makes it uncomfortable and often requires sedation or analgesia. While many new devices are being developed to attempt to address the shortfalls of conventional endoscopy (Table 1), COVID-19 has further highlighted the need for innovation in the field of flexible endoscopy. One such improvement is the use of disposable endoscopes that have already gained popularity due to concerns around duodenoscope related infection and infected biofilm in endoscope channels (Rauwers et al., 2018; Balan et al., 2019). There is also a considerable argument for a single use endoscope in reducing the running costs of departments by preventing the need for costly decontamination facilities and supplies (Larsen et al., 2020). The current pandemic has increased the awareness of IC, and the time has come to explore single use endoscopes further.

**TABLE 1:** Examples of robotic flexible endoscopy (RFE) platforms.

Device	Actuation and features	Outcome of clinical studies
Aer-O-Scope system (GI view, ramat Gan, Israel) (Pfeffer et al. (2006); Gluck et al. (2016))	Two cameras, one front viewing and second giving a 360° panoramic view that can see behind folds, disposable, tip-pulling locomotion, computer aided control, no steering or instrument channel	Cecal intubation was successful in 55/56 recruited patients (98.2%). System detected 87.6% of polyps. No mucosal damage or adverse events were reported.
		*Available on the market.*
Neo-guide endoscopy system (NeoGuide endoscopy system Inc., Los Gatos, CA, United States) (Eickhoff et al. (2007))	Electromechanical actuation of 2 independent 2 DOF segments to achieve snake - like motion, shape retention, instrument channel, 3D map of the device, computer-aided control, reusable so requires cleaning, large diameter	Cecal intubation was successful in 10 patients in the time range 24–60 min.
		No longer available on the market.
Invedosacope TMSC40 (Invendo medical GmbH, Weinheim, Germany and AMBU A/S, Copenhagen, Denmark) (Rosch et al. (2008); Groth et al. (2011); Yeung et al. (2019))	Disposable colonoscope (10 mm in diameter, with a 3.1-mm working channel), controlled electro-hydraulically by actuators placed outside the patient, operator controlled with a joystick interface, disposable, instrument channel, diameter similar to a colonoscope	Cecal intubation was successful in 98.4%, reported to be painless in 92% of patients.
		No longer available on the market.
Endotics (ERA endoscopy SRL, Peccioli, Italy) (Cosentino et al. (2009); Tumino et al. (2010); Tumino et al. (2017))	Inchworm movements, disposable, steerable tip with integrated camera and light source, computer-aided control, thin tip, no instrument channel, and procedure times longer than colonoscopy	Significantly lower patient discomfort and was also able to complete 93% of colonoscopies that were left incomplete through conventional colonoscopy
		Available on the market.
Consis medical (Beer’Sheva, Israel) (Yeung et al. (2019))	Consists of an inverted sleeve that self-propels through the colon using hydraulic aided propulsion. The sleeve is disposable, while the device head is a capsule that can be sterilised.	No available clinical studies
NaviCam^®^ (Ankon technologies co, Ltd. Wuhan, Shanghai, China) (Liao et al. (2016))	A wireless capsule endoscope that can be actuated internally by an external magnetic field.	Mean duration of examination was 25 ± 7 min. Anxiety, discomfort and pain scores (worst-best = 0–10) were 1 ± 0, 1.3 ± 0.6, and 1 ± 0 respectively.
Magnetic flexible endoscope (MFE)(STORM Lab, Leeds, United Kingdom/Nashville, TN, United States) (Martin et al. (2020))	Relies on actuation using a permanent magnet manipulated by a robot that is external to the patient; no push activation, instrument channel, large one-time robot cost and complexities around magnetic control	Currently undergoing human trials

Another avenue of improvement in endoscopy is through robotic advancements, which have the potential to reduce pain and widen the availability of procedures as they often place a lower cognitive and physical (improved ergonomics) burden on the operator and thus require less training. Robotic flexible endoscopy (RFE) can offer many advantages in general and specifically when dealing with IC related to aerosol generation. During the time of COVID-19, these include introducing physical distancing between HCW and patients (enabled as a result of teleoperation) as well as reduce the number of HCW in the endoscopy suite. In this review, we explore the impact of COVID-19 on GI endoscopy processes and how robotic flexible endoscopic platforms can be designed to minimize these impacts and improve IC mechanisms.

## Impact of COVID-19 on Flexible GI Endoscopy

### Reduction of GI Endoscopic Capacity and Its Long-Term Implication

Due to COVID-19 guidance, endoscopic procedures were significantly reduced during the pandemic to acute, urgent cases. In Europe, the volume of these procedures fell to 15% of previous capacity between February to May 2020 during the peak of the pandemic, while in North America, these levels were at 10% (Parasa et al., 2020). The National Endoscopy Database (The National Endoscopy Database, 2020) shows that endoscopic procedures fell to about 5% of normal levels in the United Kingdom. They were down from about 35,000 reported procedures per week to 1,700 for the week ending April 13^th^, 2020. When compared to 2019 levels in the United Kingdom, 51% more people were waiting for colonoscopies, 46% more for flexible sigmoidoscopies and 44% more patients were waiting for gastroscopies as of July 2020 (Cancer Research UK, 2020). While these numbers are specifically focused on the United Kingdom, a similar trend was seen worldwide in almost every national healthcare system. The statistics in this review are sometimes focused on the United Kingdom and United States as these are just a result of easily accessible data. It is estimated that for every week that screening is paused, 7,000 people are not being referred for further tests and 380 cancers are not being diagnosed through screening programs in the United Kingdom (Roberts, 2020).

Avoidable deaths due to suspended screening and treatment are expected to rise significantly with an estimated increase of more than 6,000 excess deaths in the United Kingdom and more than 33,000 excess deaths in the US (Lai et al., 2020). Endoscopic capacity has still not been restored to pre-pandemic levels (at 80% in September 2020) and with a second wave imminent, it becomes important to set the scene for future IC measures and to understand how RFE can service this need.

Current endoscopic procedures rely on PPE (enhanced during COVID-19), comprehensive room and equipment cleaning and of course air circulation (Garbey et al., 2020) which can vary for endoscopy suites. Despite concerns around patient exposure to the virus due to contaminated endoscopes, evidence suggests that reprocessing agents with viricidal activity will remove the SARS-CoV-2 virus (Kampf et al., 2020; Rai, 2020). In the past, certain bacteria were not successfully removed from duodenoscopes (Rauwers et al., 2018; Balan et al., 2019), starting a push for disposable endoscopes. There is some evidence of bronchoscope contamination (Ofstead et al., 2020), and therefore single-use endoscopes are considered safer, with potential cost savings in the long run from not having large-scale cleaning facilities on premises.

### Other Infection Control Measures

Another IC technique has been to adapt the current facilities such as the endoscopy suite to increase safety during the COVID-19 pandemic. Modified face masks or boxes have been developed as mechanical barriers (Narwani et al., 2020; Tsui, 2020). Paired with CO_2_ extractors or suction mechanism, they create negative pressure zones with the potential of eliminating (or significantly reducing) aerosols escaping the procedure site and travelling around the suite. None of the mentioned risk minimizing techniques have been quantified and the evidence around aerosol dispersion while using these methods has not been published. There is a clinical need to quantify aerosol generation during standard flexible endoscopic procedures as well as robot assisted approaches. Apart from that, there is a need for evaluation of mechanical barriers and extractors currently employed in the hospitals to provide best and uniform guidelines for clinicians working during a pandemic. Air filtration is another key aspect in reducing HCW exposure to any potential aerosols (Garbey et al., 2020).

### The Ideal RFE

A flexible robotic endoscopic platform must be able to meet certain needs during COVID-19. A teleoperated platform would allow physical distancing between the patient and HCW. A simple and easy to use robotic platform could reduce the number of people in the room allowing reallocation of staff at a crucial time and putting fewer HCW at risk during each procedure. A less painful procedure which does not require sedation would also reduce the number of people in the room. More intuitive navigation will reduce training times for future endoscopists. A RFE platform would preferably have higher degrees-of-freedom (DOF) than current commercial platforms and improved control. In addition, AI systems can be used for improved navigation and localization of endoscopes using data from both imaging and on-board sensing. This increased control and better visibility of the intestine would improve detection rates while allowing procedures such as tissue sampling and cancer removal to be performed. By reducing the force and torque exerted on the luminal wall, patient discomfort can be reduced in addition to sedation requirements thus lowering risk and recovery times. Finally, it is important to consider the environmental impacts of single-use endoscopes and using recyclable materials would be beneficial for future platforms.

## Robotic Flexible GI Endoscopy

The motivation for developing robotic platforms in endoscopy is similar to the motivation in other biomedical areas such as robotically assisted surgery. These robotic platforms allow HCW to overcome the current limitations of standard endoscopic devices for diagnostic and therapeutic procedures. These platforms can be used to improve the precision and safety of the tools thus making them more reliable and effective (Boškoski and Costamagna, 2019). The challenges for these platforms are around locomotion of endoscopes and instrument control as well as their applicability to a wide variety of clinical applications. RFE has the potential to increase safety of procedures by lowering risk of tissue damage due to human error and lower the rates of complications.

RFE has the potential to meet the needs of enhanced IC measures during COVID-19 and future pandemics. Remotely controlled devices increase the distance between patient and operator, hence less aerosol and droplet contact and reduced infection risk for HCW (Wong et al., 2004). Easy to operate robotic platforms could also help to reduce the HCWs in the room and reduce training times. It could also be extremely useful in cases where precise fine motor skills are required and cannot be met by normal human dexterity by improving the control and precision of endoscopes in robotic platforms (Lucarini et al., 2015). This will improve the speed and accuracy of procedures which will reduce adverse effects and discomfort during the procedure. Both will result in patients spending less time in the endoscopy suite.

### Advantages of RFE

With the potential advantage of lowering discomfort for patients, RFE can be used in cases where the patient is unable to tolerate conventional endoscopy or in cases where frailty and co-morbidity rule out the use of sedation during conventional endoscopy procedures. This will be achieved by having precise control over the force and torque applied to the luminal walls through force feedback. It will be hugely beneficial in cases where patients require repeated and regular procedures (e.g. surveillance in those with inflammatory bowel disease, or hereditary colorectal cancer) where using a less painful procedure will enhance surveillance uptake. It can also help to improve detection rates as more control and more comfort means better visibility. Another gap that RFE could fill is in cases where conventional endoscopes are unable to complete an examination.

While RFE is a potential solution for diagnostic endoscopy and therapeutic procedures in the GI tract, challenges remain due to the limited DOF of such platforms. Therefore, current RFE research focuses on increasing the manoeuvrability and control of the tools to enable more complex and varied interventions. Flexible endoscope manipulations common to most systems are shaft insertion and tip steering. These complex movements add difficulty in developing robot-assisted flexible endoscopes.

### Examples of RFE

Over the years many researchers have developed RFE platforms focused on various actuation and control approaches. There are several recent reviews (Li et al., 2007; Ciuti et al., 2016; Tapia-Siles et al., 2016; Li and Chiu, 2018; Boskoski and Costamagna, 2019; Ciuti et al., 2020; Visconti et al., 2020) that cover these technologies and other cutting-edge platforms in depth which is not the remit of this paper. A selection of them are listed in Table 1. The Bellowscope is another promising device not included in the table since it is not strictly a robotic platform but is a low-cost disposable endoscope. Its working principle is based on pistons and bellow actuators which are controlled by multi-DOF handheld controller (Garbin et al., 2018a; Garbin et al., 2018b; Garbin et al., 2019; Chandler et al., 2020).

Wireless endoscopes (capsules) are a great tool for diagnostic purposes and providing painless inspection of the GI tract. Introduction through the mouth and lack of wiring makes them ideal for diagnostic applications (Liao et al., 2016). The less invasive a procedure is; the less risk it carries, more so in the current climate. Currently available capsules can visualize the small bowel (such as the PillCam (Li et al., 2007)) and therefore have good diagnostic capabilities but lack therapeutic capabilities. Capsules for upper GI tract are more difficult but some devices with handheld magnetic control have shown some promise, again solely for diagnostic use. The best technical solution at the moment is the NaviCam® (Ankon Technologies Co, Ltd. Wuhan, Shanghai, China), with a wireless capsule endoscope steered magnetically inside the stomach filled with water (Jiang et al., 2019). This can be classified as a robotic solution as there is a robotic arm moving the magnetic field generator. This is in use in almost every large hospital in China. The colon capsule, however, is less successful. Without the ability to clean the stool coating the mucosa the views are limited. In addition, up to 50% of people will have a pathology requiring biopsy and therefore, many end up requiring conventional colonoscopy anyway. While capsule endoscopes would have major advantages in the case of aerosolized particles (Slawinski et al., 2015; Ciuti et al., 2016); they still have severe limitations for GI endoscopy. They lack the ability to take biopsy samples or perform therapeutic procedures. The requirements for a perfect capsule would include enhanced locomotion, location, vision, telemetry, energy, and diagnostic and therapeutic tools. A further limitation is including all these technologies in a capsule that is small enough to safely traverse the GI tract (Kwack and Lim, 2016; Singeap et al., 2016).

## Challenges for Robotic Flexible GI Endoscopy to Overcome During COVID-19

RFE can introduce physical distancing into the endoscopy units. However, one of the main challenges is related to feeding and manoeuvring the flexible endoscope. Endoscopists can be severely limited due to a lack of manoeuvrability when manually operating the endoscopes during both diagnostic and therapeutic procedures. It has been documented that during the removal of abnormal tissue (polyps) during colonoscopies, even well-experienced endoscopists can miss up to 20% of the tissue (van Rijn et al., 2006). Robotic platforms use various actuation mechanisms for endoscopes with varying levels of manoeuvrability. In fact, in many review papers, robotic endoscopes are classified based on their actuation principles which typically fall into one of the following categories: 1) magnetic, 2) electric or 3) hydraulic or pneumatic with many devices using a combination of these principles. For example: a legged robotic endoscope has recently been developed (Lee et al., 2017; Lee et al., 2019) that can be operated with an electric motor connected to reel-based mechanism that is both simple and reliable. By using soft materials for the legs, a high degree of manoeuvrability was achieved with no scratches or perforations on a porcine tissue. This is just one example of how robotic endoscopes in development can solve some of the current challenges.

Another challenge is related to operating an endoscopic instrument through the working channel, making sure that no aerosols come back from that channel. The working channel of the endoscopes can be responsible for releasing aerosols or droplets into the suite during standard operating procedure (Vavricka et al., 2010). However, very little quantifiable data exists around whether this is an issue and how big of a challenge it could be. A study that measures the aerosol levels in GI endoscopy suites during procedures would be a welcome addition to the field as well as a follow-up comparison with robotic platforms.

**FIGURE 1:**
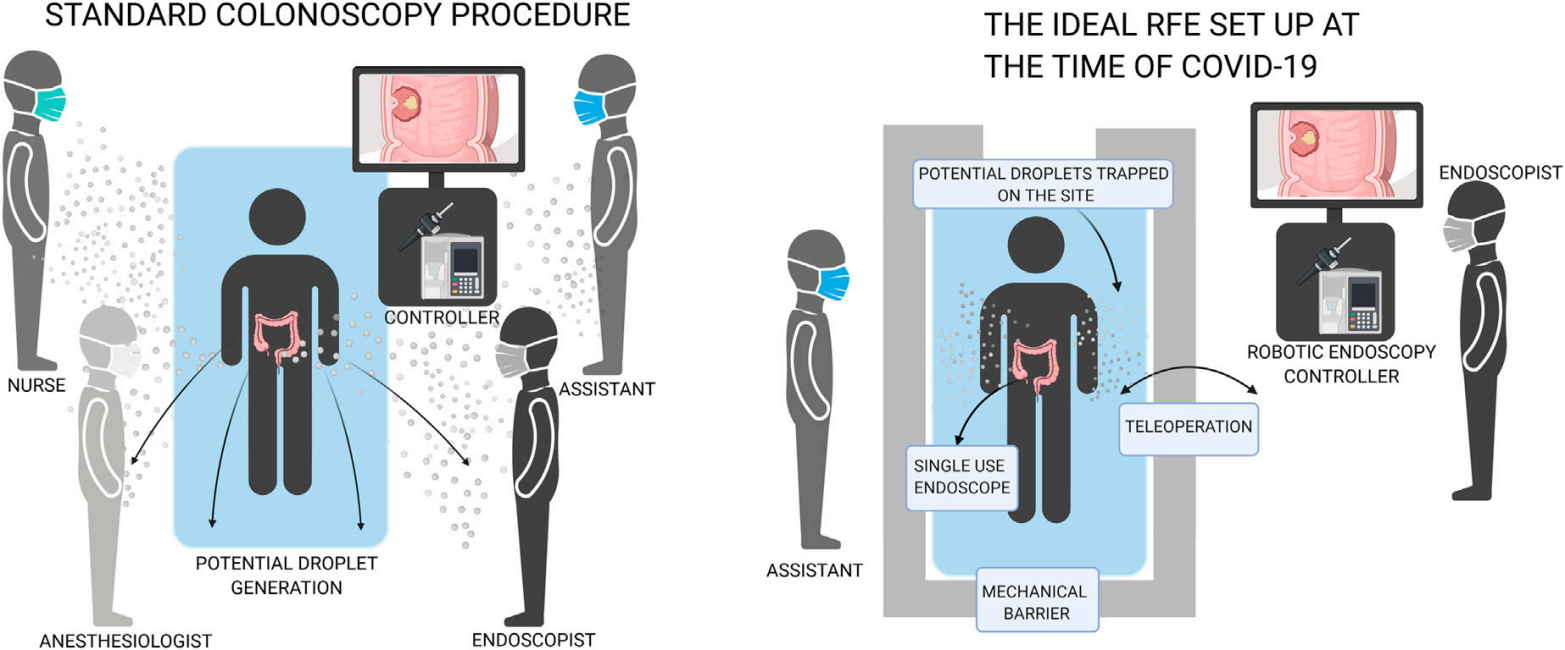
Comparison of conventional endoscopy with robotic flexible endoscopy which can increase distance and decrease number of people in the room.

Introducing robotic platforms to endoscopic systems simplifies the procedure. Teleoperation allows clinicians to control the endoscope from a safe distance or/and behind mechanical barriers, with reduced need for direct contact with the patient. Reduced discomfort means less monitoring is required and no need for additional anaesthesiologists or nursing staff, thus reducing the risk to HCW as can be seen in Figure 1.

Following evidence of transmission of infection despite decontamination, via infected biofilm in endoscopes (Rauwers et al., 2018; Balan et al., 2019), single use endoscopes are gaining popularity (Pfeffer et al., 2006; Rosch et al., 2008; Cosentino et al., 2009; Tumino et al., 2010; Groth et al., 2011; Gluck et al., 2016; Tumino et al., 2017; Yeung et al., 2019). For example: the Aer-O-Scope proposes RFE with disposable rectal introducer and supply cables for colonoscopy (Pfeffer et al., 2006; Gluck et al., 2016). Research on fully disposable endoscopes with robotic platforms should be prioritized to implement these solutions in hospitals during the COVID-19 pandemic or future airborne virus pandemics. Single use conventional endoscopes are becoming commercially available. An alternative to disposable endoscopes is tool protection (e.g. protective sheet) which can be disposed of after the procedure, followed by routine sterilisation of the remaining part. However, decontamination of endoscopy equipment is costly, and additionally places more HCW at risk of contraction of infections such as coronavirus during the decontamination process. There is evidence that some tools, such as bronchoscopes (Ofstead et al., 2020) and duodenoscopes (Rauwers et al., 2018; Balan et al., 2019), might still be contaminated with bacteria after routine sterilization. Recurrent passing of instruments down the working channel of the scope leads to damage, that damage leads to accumulation of biofilm which can become infected (Alfa and Singh, 2020; Bouiller et al., 2020; Santos et al., 2020).

## Discussion

RFE procedures have the potential to be completed with increased speed and without sedation and fewer complications, leading to a shorter recovery time, freeing space in endoscopy suites at a crucial time where endoscopic capacity has still not recovered to pre-pandemic levels. GI endoscopic procedures have been deemed high risk of contact and droplet formation with the potential to be aerosol generating. Despite little evidence as of now as to the true aerosol generating potential of GI endoscopic procedures, enhanced IC measures are likely to continue.

The ideal RFE during COVID-19 would combine teleoperation, single-use endoscopes and mechanical barriers/seals. Teleoperation allows for physical distancing between patients and HCW, single-use endoscopes would reduce the risk of contaminated scopes. A mobile device would also be extremely useful at a time when the endoscopy suite capacity is limited to make space for COVID-19 patients. RFE has significant potential in diagnostic and therapeutic endoscopy, but some challenges remain to developing the ideal pandemic-secure RFE. There is a significant need for a study to define the aerosol generation during GI endoscopy in order to tailor future guidance for both patients and HCW and maintaining capacity levels in order to avoid devastating long term implications. Even with the hope of a successful vaccine rollout, we have learned that healthcare technologies should be resilient to pandemics in general, so we believe this review will still be relevant in the future.
